# Tackling Immune Pathogenesis of COVID-19 through Molecular Pharmaceutics

**DOI:** 10.3390/pharmaceutics13040494

**Published:** 2021-04-05

**Authors:** Matteo Puccetti, Claudio Costantini, Maurizio Ricci, Stefano Giovagnoli

**Affiliations:** 1 Department of Pharmaceutical Science, University of Perugia, 06132 Perugia, Italy; maurizio.ricci@unipg.it; 2 Department of Medicine and Surgery, University of Perugia, 06132 Perugia, Italy; costacla76@gmail.com

**Keywords:** COVID-19, tryptophan metabolites, anakinra, molecular pharmaceutics

## Abstract

An increasing number of clinical studies worldwide are investigating the repurposing of antiviral, immune-modulatory, and anti-inflammatory agents to face the coronavirus disease-19 (COVID-19) pandemic. Nevertheless, few effective therapies exist to prevent or treat COVID-19, which demands increased drug discovery and repurposing efforts. In fact, many currently tested drugs show unknown efficacy and unpredictable drug interactions, such that interventions are needed to guarantee access to effective and safe medicines. Anti-inflammatory therapy has proven to be effective in preventing further injury in COVID-19 patients, but the benefit comes at a cost, as targeting inflammatory pathways can imply an increased risk of infection. Thus, optimization of the risk/benefit ratio is required in the anti-inflammatory strategy against COVID-19, which accounts for drug formulations and delivery towards regionalization and personalization of treatment approaches. In this perspective, we discuss how better knowledge of endogenous immunomodulatory pathways may optimize the clinical use of novel and repurposed drugs against COVID-19 in inpatient, outpatient, and home settings through innovative drug discovery, appropriate drug delivery systems and dedicated molecular pharmaceutics.

## 1. Introduction

Coronavirus disease-19 (COVID-19) is caused by severe acute respiratory syndrome coronavirus 2 (SARS-CoV-2), a potentially fatal clinical syndrome that involves the lower airways and leads to interstitial pneumonia in humans with hyperinflammation and respiratory dysfunction [1]. The disease is characterized by three clinical stages: (i) an asymptomatic phase, accounting for 80 to 84% of cases, (ii) a non-severe symptomatic phase, potentially evolving to a hypoxemic pneumonia or (iii) to a severe, potentially lethal disease with hypoxia, lung infiltrates, and ultimate acute respiratory distress syndrome (ARDS) [1]. Drugs that inhibit key components of the coronavirus infection lifecycle have been repurposed in COVID-19 therapy [2], with the support of proper cheminformatic tools as well to expedite the identification of potential candidates and treatment modalities [3,4,5,6,7]. The mild to severe progression of COVID-19 depends on the extent and features of the individual immune response to the virus. Indeed, significant specific or non-specific organ damages can stem from the host’s own cellular and humoral immune responses to the infection. Therefore, COVID-19 pathogenesis is the result of a cascade of events starting from high levels of circulating proinflammatory cytokines that can evolve to a cytokine storm, responsible for non-specific inflammatory cell infiltration and contributing to downstream pulmonary and interstitial tissue damage [8]. Such conditions can quickly develop into ARDS with lethal consequences.

Thus, immunomodulatory agents capable of restraining or suppressing such progressions are logical candidates in COVID-19 therapy. Not surprisingly, interventions based on non-steroidal anti-inflammatory drugs (NSAIDs), glucocorticoids, intravenous immune globulins, immunosuppressants, chloroquine/hydroxychloroquine, IL-1 antagonists, IL-6R monoclonal antibodies, TNF inhibitors, and Janus kinase (JAK) inhibitors have been capable of relieving severe disease conditions in COVID-19 patients [9,10,11]. Nevertheless, a dark side in targeting inflammatory pathways exists, owing to a higher risk of opportunistic infections. In particular, the benefit of the use of glucocorticoids, IL-6 and Janus kinase inhibitors is likely outweighed by adverse effects, such as significantly increased risk of mortality and secondary infections [9]. However, an optimal risk/benefit ratio balance could be ideally granted by immunomodulatory agents capable of delivering anti-inflammatory input at the target organs, while preserving the immune system’s capacity to respond to pathogen invasions. This requires a better knowledge of immunoregulatory pathways underlying the homeostatic regulation of inflammation in the diseased organs to efficiently respond to infection while preventing damage.

Herein, we first describe current anti-inflammatory strategies, and then provide an example of how a better knowledge of inflammatory/anti-inflammatory endogenous pathways may optimize the clinical use of anti-inflammatory therapy in COVID-19 through innovative drug discovery, the selection of the appropriate administration route, drug delivery systems, and dedicated molecular pharmaceutics.

## 2. Current Anti-Inflammatory Approaches

The dramatic urgence of restraining the SARS-CoV-2 pandemic and preventing COVID-19 severity has granted several anti-inflammatory drugs an off-label use or a fast track to clinical trials (The Pharmaceutical Journal, February 2021; Online: doi: 10.1211/PJ.2021.20208126, last updated 24 February 2021, [12]). These include corticosteroids (i.e., dexamethasone), cytokines (i.e., interferons), drugs that interfere with cytokine activities (i.e., tocilizumab and sarilumab, which block IL-6 activity, canakinumab and anakinra, which block IL-1, or infliximab and adalimumab, which block TNFα) and signaling pathways (i.e., baricitinib and ruxolitinib, and JAK1/2 inhibitors).

Despite initial concerns [13], clinical evidence supports the efficacy of corticosteroids in the treatment of severe COVID-19 patients. Retrospective observational studies indicated that severe COVID-19 patients had a more favorable evolution if treated with corticosteroids [14], although other studies found either no effect [15] or a delayed healing [16] in hospitalized patients. A meta-analysis of seven randomized clinical trials, including 1703 hospitalized, critically ill patients, reported a lower 28-day all-cause mortality upon administration of systemic corticosteroids compared to usual care or placebo [17]. Currently, dexamethasone is strongly recommended for hospitalized patients requiring oxygen delivery through a high-flow device, non-invasive ventilation, invasive mechanical ventilation or extracorporeal membrane oxygenation (https://www.covid19treatmentguidelines.nih.gov/therapeutic-management/, last updated 11 February 2021).

The use of NSAIDs also initially received concerns for COVID-19 treatment [18]. Several observational studies, however, have shown that NSAIDs are not associated with mortality or severity of disease [19,20,21,22,23,24] and their potential use in the treatment of COVID-19 has been proposed [25,26]. A recent retrospective analysis of data in Electronic Health Records (EHRs) to identify drugs with the potential to be repurposed to treat COVID-19 has identified, among others, ibuprofen as associated with a lower risk for COVID-19 outcomes [27]. This is in line with a previous study analyzing EHRs in six Eastern Massachusetts hospitals that identified a significant association between ibuprofen and diminished risk for hospitalization [28]. Currently ongoing clinical trials evaluating the efficacy and safety of ibuprofen will provide definite evidence for the potential clinical use of NSAIDs in COVID-19.

Alongside corticosteroids, monoclonal antibodies directed towards cytokine receptors look promising to decrease hyperinflammation. In fact, a recent preprint report on the Randomized Evaluation of COVID-19 Therapy (RECOVERY) trial, showed that tocilizumab, a humanized antibody binding the IL-6 receptor, was effective in hospitalized patients with hypoxia and systemic inflammation and the benefits were present also in patients receiving systemic corticosteroids [29]. In addition, a retrospective analysis of data extracted from the RECOVERY study and seven previous randomized controlled trials confirmed a tocilizumab associated reduction of 28-day mortality [29]. Recently, the results from the REMAP-CAP trial have been published [30] demonstrating that not only tocilizumab, but also the other IL-6 receptor antagonist, sarilumab, improved outcomes, and this occurred also in patients treated with glucocorticoids.

The RECOVERY trial is also expected to provide results on colchicine, an alkaloid with anti-inflammatory effects, that may bear potential therapeutic efficacy in COVID-19 [31].

Another anti-inflammatory strategy includes inhibitors of signaling pathways mediating cytokine activity, such as the JAK/STAT pathway [32]. The results of the ACTT-2 trial in hospitalized adults with COVID-19 indicate that baricitinib plus remdesivir was superior to remdesivir alone in the primary outcome, i.e., the time to recovery, and the key secondary outcome, i.e., the clinical status at day 15 [33], thus showing promise for the use of JAK inhibitors, including not only baricitinib but also ruxolinitib and tofacitinib, in the treatment of COVID-19. However, evidence for the potential combination with corticosteroids remains to be provided.

Overall, targeting inflammation is a worthwhile strategy to combat COVID-19 and prevent disease severity. Nevertheless, even though promising results are emerging from clinical trials, systemic administration of anti-inflammatory drugs exposes the patients to additional risks and is associated with low compliance, especially in the case of biotechnological drugs. Therefore, specific approaches affording a localized action should be preferred to improve the efficacy/safety profile, as we will illustrate in the following sections for two endogenous pathways of immunomodulation.

## 3. The Inflammasome Pathway

The interferon and the NF-κB pathways have been recognized as being among the primary activated signaling cascades in SARS-CoV-2 infection [34], producing high IL-1β, TNF-α, and IL-6 serum and tissue levels [35,36]. Albeit being potentially protective by promoting CD8+ T cells and phagocytes responses against infected cells and the production of virus-specific antibodies, when highly expressed, these cytokines may contribute to COVID-19 pathogenesis for their role in the induction of the cytokine storm [37].

Notably, only the IL-1 pathway seems to affect the phases preceding the respiratory function, nadir [38], such that early blockade of the IL-1 receptor (IL-1R) was effective in treating acute hyperinflammatory respiratory failure in COVID-19 patients [39,40,41,42,43,44]. By causing the release of IL-1α and β, the activated IL-1 signaling pathway is considered a fundamental bridge between inflammasome disreactivity, mostly driven by dysfunctional NLRP3 activity and lung inflammation [45], including in acute lung injury after respiratory viral infections [46].

Upon different stimuli, inflammasomes lead to the synthesis of IL-1β by recruiting caspase-1 that cleaves the pro-IL-1β precursor to give the active form.

Being that inflammasome activity is dysregulated in COVID-19 [47,48,49], following the infection, alveolar macrophages secrete TNF-α and IL-1β, giving rise to cell death, damage, and NLRP3 activation that trigger the acute proinflammatory cascade. Furthermore, angiotensin-converting enzyme 2 signaling has also been implicated in NLRP3 activation [48].

In a subsequent phase, such initially localized inflammatory events spread to the vasculature, producing leakage, edema, and pneumonia, typical of COVID-19 [48].

The coronavirus tolerance observed in bats has been associated to a dampened transcriptional priming of NLRP3 [50], which confirms that targeting the NLRP3/IL-1β pathway is a successful strategy in COVID-19. Several clinical studies seem to confirm this, by showing the efficacy of IL-1R receptor antagonists (IL-1Ra), such as anakinra, against COVID-19, even in patients with co-morbidities and combined with antiviral drugs [39,40,41,42,43,44].

Anakinra is a recombinant non-glycosylated form of IL-1Ra showing higher affinity for IL-1R1 than that for IL-1 itself [51]. Anakinra (Kineret^®^) is a drug marketed in 2001 for the treatment of rheumatoid arthritis by subcutaneous administration of 100 mg daily and, more recently, of cryopyrin-associated periodic syndromes and systemic-onset juvenile idiopathic arthritis, and is widely used off-label [52]. Its therapeutic potential derives from the ability to prevent IL-1α and IL-1β driven inflammation. Anakinra clinical use is supported by a recognized safety and the evidence in murine lung and human bronchial epithelial cells of a potent inhibition of pathogenic NLRP3 activation and concurrent IL-1β, TNF-α and IL-6 suppression [53].

**Figure 1:**
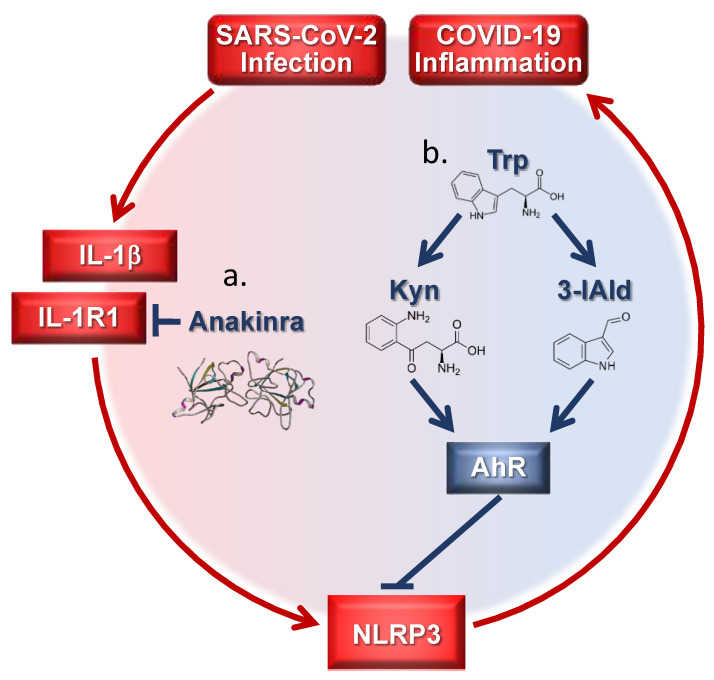
Targeting inflammatory pathways in coronavirus disease-19 (COVID-19) by tryptophan (trp) metabolites and Anakinra. (**a**) Anakinra inhibits NLRP3 by blocking IL-1R1 activation; (**b**) endogenous trp metabolites target AhR downregulating the NLRP3 pathway. Kyn = kynurenine; 3-IAld = Indole-3-carboxaldehyde.

Together, these studies suggest that modulating NLRP3 or IL-1R1 related inflammatory responses could be a successful therapy in COVID-19 (Figure 1). It is worth mentioning that canakinumab, an antibody targeting IL-1β, has also been reported to improve outcomes [54,55,56]. However, the phase III CAN-COVID trial in hospitalized patients did not meet the primary endpoint, i.e., greater chance of survival without the need for invasive mechanical ventilation, and the key secondary endpoint of reduced COVID-19 mortality (https://www.novartis.com/news/media-releases/novartis-provides-update-can-covid-trial-hospitalized-patients-covid-19-pneumonia-and-cytokine-release-syndrome-crs; accessed 26 March 2021). Our recent observations that anakinra is capable of inhibiting NLRP3 and inducing autophagy by a mechanism independent of the known activity on IL-1R1 that involves a xenobiotic sensing pathway coupling mitochondrial redox balance to autophagy (manuscript submitted) suggest that the activity of anakinra is more complex than previously thought and may help to reconcile the results of the clinical trials.

## 4. The Xenobiotic Pathway

Interferons (IFNs), either alone or combined with antiviral agents, are currently being explored for the treatment of COVID-19, owing to their role in innate immunity. Type I IFNs (alpha and beta) are secreted upon viral infection and are known to have antiviral activity against coronaviruses, which explains the considerable number of current clinical studies listed on ClinicalTrials.gov (US National Library of Medicine, 2020). IFNs are known to shift tryptophan (trp) catabolism away from serotonin toward kynurenines [57] via the enzyme indoleamine 2, 3-dioxygenase (IDO)1. IDO1 together with tryptophan-2, 3-dioxygenase have been related to inflammatory diseases, cancer, diabetes, and mental disorders in light of their regulatory role in kynurenine production in the trp metabolic pathway [58,59,60]. IDO1 has an important role in preserving immune tolerance and homeostasis in the lungs [61,62]. Therefore, it is not surprising that the IDO1/kynurenine pathway is upregulated in COVID-19 due to the rise in pro-inflammatory cytokines [63]. This implies that more than IDO1 mimetics, alternative pathways of trp utilization could be exploited for tolerance induction in the lung.

The Aryl Hydrocarbon Receptor (AhR) is a ubiquitous ligand-activated transcription factor mainly expressed in barrier organs, such as the lungs, skin, liver, and gut [64]. Particularly in these organs, AhR exerts a fundamental regulation of the immune response and the maintenance of mucosal homeostasis [65]. Albeit still debated, the increasing body of literature connects AhR signaling to the preservation of lung health [66]. Such an AhR role may help contrasting lung pathogens by sensing virulence factors and promoting the subsequent recruitment of inflammatory cells [67]. Activation of AhR by CoV may change disease phenotypic features based on time after infection but also on diet and environmental factors [68].

Evidence supporting the role of AhR in lung physiology, including negative NLRP3 regulation [69], could provide new COVID-19 therapeutic opportunities based on the AhR and/or other xenobiotic receptor biological functions (Figure 1). The AhR senses a wide variety of agonists, typically hydrophobic in nature, of either exogenous or endogenous origin [64], including metabolites produced by microbes [70]. However, it must be kept in mind that AhR biology relates to ligand nature, environment, and disease [64].

As a proof-of-concept that properly targeting AhR with an endogenous metabolite may result in beneficial effects, our preliminary observations indicate that local delivery of a microbial metabolite, administered either orally via microparticle encapsulation or via lung in a spray-dried formulation, could alleviate inflammation in mice with respiratory infection and inflammation (Puccetti et al. manuscript in preparation). Thus, the proper targeting of AhR in the lung alleviates the inflammatory response during infection.

## 5. Concluding Remarks

The requirements for drug formulations have increased significantly in recent decades, boosted by the current industry trends towards regionalization and personalization of treatment approaches. This trend is what demanded for the optimal delivery of anti-inflammatory agents in COVID-19, given the need for fine balancing benefits and risks. New formulations and techniques for the extended and precise dosing of medicines are now in place, such as spray-drying to produce enteric microparticles for local intestine release and inhalable dry powders for lung delivery [71]. Inhaled products can grant localization of therapeutic action, enabling dose reduction and lowering the risk of off-target effects [72], and are credited as an optimal delivery form for proteins and peptides [73]. Inhaled peptides have been already marketed or are under clinical development [71]. Likewise, despite the gut adverse environment, novel emerging formulations show promises for protein oral delivery [74]. Anakinra comes in prefilled syringes for subcutaneous injection at an individual dose of 100 mg/0.67 mL/day. Although highly bioavailable (95%) [75], reaching maximum plasma levels in 3–7 h with a terminal half-life of 6–8 h, the current once-a-day subcutaneous injection of anakinra is relatively low compliant and may show lower efficacy when delivered systemically in lung infections, such as in the case of COVID-19. Despite the safety profile and low toxicity, even in patients with asthma history, injection site reactions in addition to self-medication issues can result in patient discomfort that discourages this regimen. Thus, the high compliance of the lung and oral routes and the existence of enabling technologies for fast translation to the clinic make the oral and pulmonary delivery of anakinra a very attractive approach in COVID-19 therapy. Indeed, inhalation could be a pivotal approach against COVID-19, since the lungs represent the main infection site, and thus a therapeutic target, as even confirmed by in silico predictive tools [76,77]. The well-known and above-mentioned advantages of inhaled drugs, particularly in the form of dry powders, could be of great benefit for drugs like anakinra, justifying the likely higher cost of production compared to the injectable form. In this regard, considering the cost of the protein drug, dose reduction compared to Kineret^®^ may partially counterbalance the above-mentioned higher expenses of the pulmonary products. Moreover, embedding the drug into a solid form extends the shelf life of the product, especially as far as biotechnological drugs are concerned, increasing its market value.

Similar to what was observed with the AhR-ligand formulations [78] (Puccetti et al., manuscript in preparation), our own ongoing project is in place with the expectation to optimize both the therapeutic efficacy of anakinra and the patient’s compliance. Thus, molecular pharmaceutics of repurposed and novel drugs may generate essential information useful for the development of anti-inflammatory-based drug discovery and delivery strategies in COVID-19.

In this regard, insightful investigation of immunological regulatory pathways has led to the identification of novel selective biologicals and small molecule drugs that have enabled tremendous advances in the treatment of chronic inflammatory diseases and tumor therapy [79].

**Figure 2:**
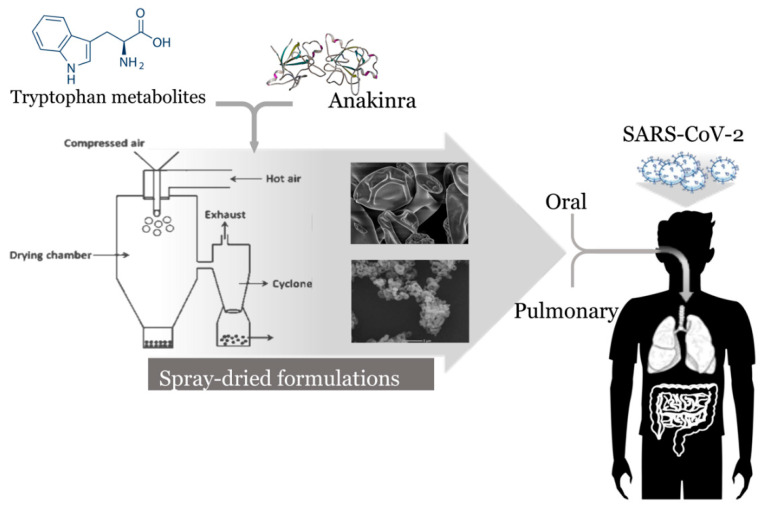
Molecular pharmaceutics of tryptophan metabolites and anakinra in the treatment of COVID-19. The pulmonary route is preferred for enhanced local effect and dose reduction. The oral route is also considered as an alternative, highly compliant and cost-effective approach.

The challenge ahead is to optimize the clinical use of biologicals to target inflammatory pathways in COVID-19 through novel drug delivery platforms and dedicated molecular pharmaceutics (Figure 2).

The recent development of inhaled forms of remdesivir for protecting and treating the respiratory mode of SARS-CoV-2 infection [80] and the relevant number of new or repurposed inhaled drugs under clinical development (Table 1) emphasize how molecular pharmaceutics, by allowing more widely available early-stage intervention methods to non-hospitalized patients, could significantly lessen symptoms before they become potentially life-threatening, lower costs, and reduce transmission.

## Figures and Tables

**Table 1:** Ongoing clinical trials of new and repurposed inhaled drugs in COVID-19 (ClinicalTrials.gov, accessed Oct. 2020, not intended to be exhaustive).

Location	Study Title	Development Stage
U.S.	Safety, tolerability and pharmacokinetics of inhaled nanoparticle formulation of remdesivir (GS-5734) and NA-831 (NEUROSIVIR)	Phase I
Switzerland	Inhaled aviptadil for the prevention of COVID-19 related ARDS	Phase I
U.S.	An experiment to evaluate the safety of agenT-797 in COVID-19 patients with severe difficulty breathing.	Phase I
U.S.	A study to evaluate the safety, tolerability and pharmacokinetics of orally inhaled aerosolized hydroxychloroquine sulfate in healthy adult volunteers	Phase I
U.S.	Study in participants with early stage coronavirus disease 2019 (COVID-19) to evaluate the safety, efficacy, and pharmacokinetics of remdesivir administered by inhalation	Phase I/II
France	Efficacy of captopril in COVID-19 patients with severe acute respiratory syndrome (SARS) cov-2 pneumonia (CAPTOCOVID)	Phase II
Egypt	Efficacy of aerosol combination therapy of 13 cis retinoic acid and captopril for treating COVID-19 patients via indirect inhibition of transmembrane protease, serine 2 (TMPRSS2)	Phase II
U.K.	Steroids in COVID-19 study (STOIC)	Phase II
U.K.	Inhaled anti-viral (SNG001) for SARS-cov-2 (COVID-19) infection	Phase II
Korea	A trial of ciclesonide in adults with mild-to-moderate COVID-19	Phase II
Egypt	Efficacy and safety of drug combination therapy of isotretinoin and some antifungal drugs as a potential aerosol therapy for COVID-19: an innovative therapeutic approach COVID-19.	Phase II
Qatar	Inhaled iloprost for suspected COVID-19 respiratory failure (ILOCOVID)	Phase II
U.K., Romania, Ukraine, Moldova	TD-0903 for ALI associated with COVID-19	Phase II
Egypt	Aerosol combination therapy of all-trans retinoic acid and isotretinoin as a novel treatment for inducing neutralizing antibodies in COVID -19 infected patients better than vaccine: an innovative treatment (Antibodies)	Phase II
Canada	Inhaled ciclesonide for outpatients with COVID-19 (CONTAIN)	Phase II/III
Iran	Evaluation of efficacy of levamisole and formoterol+budesonide in treatment of COVID-19	Phase II/III
Russia	An open randomized study of dalargin effectiveness in patients with severe and critical manifestations of SARS-COVID-19	Phase III
U.S.	A study of the safety and efficacy of ciclesonide in the treatment of non-hospitalized COVID-19 patients	Phase III
U.S.	Dornase alfa for ARDS in patients with SARS-cov-2 (DORNASESARS2)	Phase III
Spain	Inhaled corticosteroid treatment of COVID19 patients with pneumonia	Phase IV
Argentina	Nebulized heparin in severe acute respiratory syndrome COVID-19 (NEBUHEPA)	Phase IV
China	Evaluation of Ganovo (danoprevir) combined with ritonavir in the treatment of SARS-cov-2 infection	Phase IV
U.S.	Valproate alone or in combination with quetiapine for severe COVID-19 pneumonia with agitated delirium	Phase IV
Belgium	Sargramostim in patients with acute hypoxic respiratory failure due to COVID-19 (SARPAC)	Phase IV

## References

[1] Guan W.J., Ni Z.Y., Hu Y., Liang W.H., Ou C.Q., He J.X., Liu L., Shan H., Lei C.L., Hui D.S.C. (2020). Clinical Characteristics of Coronavirus Disease 2019 in China. N. Engl. J. Med..

[2] Sohag A.A.M., Hannan M.A., Rahman S., Hossain M., Hasan M., Khan M.K., Khatun A., Dash R., Uddin M.J. (2020). Revisiting potential druggable targets against SARS-CoV-2 and repurposing therapeutics under preclinical study and clinical trials: A comprehensive review. Drug Dev. Res..

[3] Ojha P.K., Kar S., Krishna J.G., Roy K., Leszczynski J. (2021). Therapeutics for COVID-19: From computation to practices-where we are, where we are heading to. Mol. Divers.

[4] Tejera E., Munteanu C.R., Lopez-Cortes A., Cabrera-Andrade A., Perez-Castillo Y. (2020). Drugs Repurposing Using QSAR, Docking and Molecular Dynamics for Possible Inhibitors of the SARS-CoV-2 M(pro) Protease. Molecules.

[5] Egieyeh S., Egieyeh E., Malan S., Christofells A., Fielding B. (2021). Computational drug repurposing strategy predicted peptide-based drugs that can potentially inhibit the interaction of SARS-CoV-2 spike protein with its target (humanACE2). PLoS ONE.

[6] Wang J. (2020). Fast Identification of Possible Drug Treatment of Coronavirus Disease-19 (COVID-19) through Computational Drug Repurposing Study. J. Chem. Inf. Model.

[7] Singh T.U., Parida S., Lingaraju M.C., Kesavan M., Kumar D., Singh R.K. (2020). Drug repurposing approach to fight COVID-19. Pharmacol. Rep..

[8] Chen G., Wu D., Guo W., Cao Y., Huang D., Wang H., Wang T., Zhang X., Chen H., Yu H. (2020). Clinical and immunological features of severe and moderate coronavirus disease 2019. J. Clin. Invest..

[9] Zhang W., Zhao Y., Zhang F., Wang Q., Li T., Liu Z., Wang J., Qin Y., Zhang X., Yan X. (2020). The use of anti-inflammatory drugs in the treatment of people with severe coronavirus disease 2019 (COVID-19): The Perspectives of clinical immunologists from China. Clin. Immunol..

[10] Felsenstein S., Herbert J.A., McNamara P.S., Hedrich C.M. (2020). COVID-19: Immunology and treatment options. Clin. Immunol..

[11] Ucciferri C., Vecchiet J., Falasca K. (2020). Role of monoclonal antibody drugs in the treatment of COVID-19. World J. Clin. Cases.

[12] Romani L., Tomino C., Puccetti P., Garaci E. (2020). Off-label therapy targeting pathogenic inflammation in COVID-19. Cell Death Discov..

[13] Prescott H.C., Rice T.W. (2020). Corticosteroids in COVID-19 ARDS: Evidence and Hope During the Pandemic. JAMA.

[14] Pascual Pareja J.F., Garcia-Caballero R., Soler Rangel L., Vazquez-Ronda M.A., Roa Franco S., Navarro Jimenez G., Moreno Palanco M.A., Gonzalez-Ruano P., Lopez-Menchaca R., Ruiz-Seco P. (2021). Effectiveness of glucocorticoids in patients hospitalized for severe SARS-CoV-2 pneumonia. Med. Clin..

[15] Andersen K.M., Mehta H.B., Palamuttam N., Ford D., Garibaldi B.T., Auwaerter P.G., Segal J., Alexander G.C. (2021). Association Between Chronic Use of Immunosuppresive Drugs and Clinical Outcomes from Coronavirus Disease 2019 (COVID-19) Hospitalization: A Retrospective Cohort Study in a Large US Health System. Clin. Infect. Dis..

[16] D’Ardes D., Pontolillo M., Esposito L., Masciarelli M., Boccatonda A., Rossi I., Bucci M., Guagnano M.T., Ucciferri C., Santilli F. (2020). Duration of COVID-19: Data from an Italian Cohort and Potential Role for Steroids. Microorganisms.

[17] Sterne J.A.C., Murthy S., Diaz J.V., Slutsky A.S., Villar J., Angus D.C., Annane D., Azevedo L.C.P., Berwanger O., The WHO Rapid Evidence Appraisal for COVID-19 Therapies (REACT) Working Group (2020). Association Between Administration of Systemic Corticosteroids and Mortality Among Critically Ill Patients With COVID-19: A Meta-analysis. JAMA.

[18] Fang L., Karakiulakis G., Roth M. (2020). Are patients with hypertension and diabetes mellitus at increased risk for COVID-19 infection?. Lancet Respir. Med..

[19] Park J., Lee S.H., You S.C., Kim J., Yang K. (2021). Non-steroidal anti-inflammatory agent use may not be associated with mortality of coronavirus disease 19. Sci. Rep..

[20] Wong A.Y., MacKenna B., Morton C.E., Schultze A., Walker A.J., Bhaskaran K., Brown J.P., Rentsch C.T., Williamson E., Drysdale H. (2021). Use of non-steroidal anti-inflammatory drugs and risk of death from COVID-19: An OpenSAFELY cohort analysis based on two cohorts. Ann. Rheum. Dis..

[21] Abu Esba L.C., Alqahtani R.A., Thomas A., Shamas N., Alswaidan L., Mardawi G. (2021). Ibuprofen and NSAID Use in COVID-19 Infected Patients Is Not Associated with Worse Outcomes: A Prospective Cohort Study. Infect. Dis. Ther..

[22] Kragholm K., Gerds T.A., Fosbol E., Andersen M.P., Phelps M., Butt J.H., Ostergaard L., Bang C.N., Pallisgaard J., Gislason G. (2020). Association Between Prescribed Ibuprofen and Severe COVID-19 Infection: A Nationwide Register-Based Cohort Study. Clin. Transl. Sci..

[23] Chandan J.S., Zemedikun D.T., Thayakaran R., Byne N., Dhalla S., Acosta-Mena D., Gokhale K.M., Thomas T., Sainsbury C., Subramanian A. (2020). Non-steroidal anti-inflammatory drugs and susceptibility to COVID-19. Arthritis Rheumatol..

[24] Rinott E., Kozer E., Shapira Y., Bar-Haim A., Youngster I. (2020). Ibuprofen use and clinical outcomes in COVID-19 patients. Clin. Microbiol. Infect..

[25] Kelleni M.T. (2021). Early use of non-steroidal anti-inflammatory drugs in COVID-19 might reverse pathogenesis, prevent complications and improve clinical outcomes. Biomed. Pharmacother..

[26] Prasher P., Sharma M., Gunupuru R. (2021). Targeting cyclooxygenase enzyme for the adjuvant COVID-19 therapy. Drug Dev. Res..

[27] Bejan C.A., Cahill K.N., Staso P.J., Choi L., Peterson J.F., Phillips E.J. (2021). DrugWAS: Leveraging drug-wide association studies to facilitate drug repurposing for COVID-19. Med. Rxiv..

[28] Castro V.M., Ross R.A., McBride S.M., Perlis R.H. (2020). Identifying common pharmacotherapies associated with reduced COVID-19 morbidity using electronic health records. Med. Rxiv..

[29] Horby P.W., Pessoa-Amorim G., Peto L., Brightling C.E., Sarkar R., Thomas K., Jeebun V., Ashish A., Tully R., Chadwick D. (2021). Tocilizumab in patients admitted to hospital with COVID-19 (RECOVERY): Preliminary results of a randomised, controlled, open-label, platform trial. Med. Rxiv..

[30] Investigators R.-C., Gordon A.C., Mouncey P.R., Al-Beidh F., Rowan K.M., Nichol A.D., Arabi Y.M., Annane D., Beane A., van Bentum-Puijk W. (2021). Interleukin-6 Receptor Antagonists in Critically Ill Patients with Covid-19. N. Engl. J. Med..

[31] Chiu L., Chow R., Chiu N., Lo C.-H., Aggarwal R., Lee J., Choi Y.-G., Lam H., Prsic E.H., Shin H.J. (2021). Colchicine use in patients with COVID-19: A systematic review and meta-analysis. Med. Rxiv..

[32] Luo W., Li Y.X., Jiang L.J., Chen Q., Wang T., Ye D.W. (2020). Targeting JAK-STAT Signaling to Control Cytokine Release Syndrome in COVID-19. Trends Pharmacol. Sci..

[33] Kalil A.C., Patterson T.F., Mehta A.K., Tomashek K.M., Wolfe C.R., Ghazaryan V., Marconi V.C., Ruiz-Palacios G.M., Hsieh L., Kline S. (2021). Baricitinib plus Remdesivir for Hospitalized Adults with Covid-19. N. Engl. J. Med..

[34] Gordon D.E., Jang G.M., Bouhaddou M., Xu J., Obernier K., White K.M., O’Meara M.J., Rezelj V.V., Guo J.Z., Swaney D.L. (2020). A SARS-CoV-2 protein interaction map reveals targets for drug repurposing. Nature.

[35] Huang C., Wang Y., Li X., Ren L., Zhao J., Hu Y., Zhang L., Fan G., Xu J., Gu X. (2020). Clinical features of patients infected with 2019 novel coronavirus in Wuhan, China. Lancet.

[36] Qin C., Zhou L., Hu Z., Zhang S., Yang S., Tao Y., Xie C., Ma K., Shang K., Wang W. (2020). Dysregulation of Immune Response in Patients with Coronavirus 2019 (COVID-19) in Wuhan, China. Clin. Infect. Dis..

[37] Song P., Li W., Xie J., Hou Y., You C. (2020). Cytokine storm induced by SARS-CoV-2. Clin. Chim. Acta.

[38] Jamilloux Y., Henry T., Belot A., Viel S., Fauter M., El Jammal T., Walzer T., Francois B., Seve P. (2020). Should we stimulate or suppress immune responses in COVID-19? Cytokine and anti-cytokine interventions. Autoimmun. Rev..

[39] Cavalli G., de Luca G., Campochiaro C., Della-Torre E., Ripa M., Canetti D., Oltolini C., Castiglioni B., Tassan Din C., Boffini N. (2020). Interleukin-1 blockade with high-dose anakinra in patients with COVID-19, acute respiratory distress syndrome, and hyperinflammation: A retrospective cohort study. Lancet Rheumatol..

[40] Cauchois R., Koubi M., Delarbre D., Manet C., Carvelli J., Blasco V.B., Jean R., Fouche L., Bornet C., Pauly V. (2020). Early IL-1 receptor blockade in severe inflammatory respiratory failure complicating COVID-19. Proc. Natl. Acad. Sci. USA.

[41] Mehta P., Cron R.Q., Hartwell J., Manson J.J., Tattersall R. (2020). Intravenous anakinra for cytokine storm syndromes—Authors’ reply. Lancet Rheumatol..

[42] Iglesias-Julian E., Lopez-Veloso M., de-la-Torre-Ferrera N., Barraza-Vengoechea J.C., Delgado-Lopez P.D., Colazo-Burlato M., Ubeira-Iglesias M., Montero-Baladia M., Lorenzo-Martin A., Minguito-de-la-Iglesia J. (2020). High dose subcutaneous Anakinra to treat acute respiratory distress syndrome secondary to cytokine storm syndrome among severely ill COVID-19 patients. J. Autoimmun..

[43] Huet T., Beaussier H., Voisin O., Jouveshomme S., Dauriat G., Lazareth I., Sacco E., Naccache J.M., Bezie Y., Laplanche S. (2020). Anakinra for severe forms of COVID-19: A cohort study. Lancet Rheumatol..

[44] Dimopoulos G., de Mast Q., Markou N., Theodorakopoulou M., Komnos A., Mouktaroudi M., Netea M.G., Spyridopoulos T., Verheggen R.J., Hoogerwerf J. (2020). Favorable Anakinra Responses in Severe Covid-19 Patients with Secondary Hemophagocytic Lymphohistiocytosis. Cell Host Microbe.

[45] Scambler T., Holbrook J., Savic S., McDermott M.F., Peckham D. (2018). Autoinflammatory disease in the lung. Immunology.

[46] Schmitz N., Kurrer M., Bachmann M.F., Kopf M. (2005). Interleukin-1 is responsible for acute lung immunopathology but increases survival of respiratory influenza virus infection. J. Virol..

[47] Van den Berg D.F., Te Velde A.A. (2020). Severe COVID-19: NLRP3 Inflammasome Dysregulated. Front Immunol..

[48] Freeman T.L., Swartz T.H. (2020). Targeting the NLRP3 Inflammasome in Severe COVID-19. Front Immunol..

[49] Yap J.K.Y., Moriyama M., Iwasaki A. (2020). Inflammasomes and Pyroptosis as Therapeutic Targets for COVID-19. J. Immunol..

[50] Ahn M., Anderson D.E., Zhang Q., Tan C.W., Lim B.L., Luko K., Wen M., Chia W.N., Mani S., Wang L.C. (2019). Dampened NLRP3-mediated inflammation in bats and implications for a special viral reservoir host. Nat. Microbiol..

[51] Cavalli G., Dinarello C.A. (2019). Corrigendum: Anakinra Therapy for Non-cancer Inflammatory Diseases. Front Pharmacol..

[52] Dinarello C.A. (2019). The IL-1 family of cytokines and receptors in rheumatic diseases. Nat. Rev. Rheumatol..

[53] Iannitti R.G., Napolioni V., Oikonomou V., de Luca A., Galosi C., Pariano M., Massi-Benedetti C., Borghi M., Puccetti M., Lucidi V. (2016). IL-1 receptor antagonist ameliorates inflammasome-dependent inflammation in murine and human cystic fibrosis. Nat. Commun..

[54] Ucciferri C., Auricchio A., di Nicola M., Potere N., Abbate A., Cipollone F., Vecchiet J., Falasca K. (2020). Canakinumab in a subgroup of patients with COVID-19. Lancet Rheumatol..

[55] Katia F., Myriam D.P., Ucciferri C., Auricchio A., di Nicola M., Marchioni M., Eleonora C., Emanuela S., Cipollone F., Vecchiet J. (2021). Efficacy of canakinumab in mild or severe COVID-19 pneumonia. Immun. Inflamm. Dis..

[56] Generali D., Bosio G., Malberti F., Cuzzoli A., Testa S., Romanini L., Fioravanti A., Morandini A., Pianta L., Giannotti G. (2020). Canakinumab as treatment for COVID-19-related pneumonia: A prospective case-control study. Internation. J. Infect. Dis. IJID Off. Publ. Internation. Soc. Infect. Dis..

[57] Wichers M.C., Koek G.H., Robaeys G., Verkerk R., Scharpe S., Maes M. (2005). IDO and interferon-alpha-induced depressive symptoms: A shift in hypothesis from tryptophan depletion to neurotoxicity. Mol. Psychiatry.

[58] Badawy A.A. (2017). Kynurenine Pathway of Tryptophan Metabolism: Regulatory and Functional Aspects. Int. J. Tryptophan Res..

[59] Comai S., Bertazzo A., Brughera M., Crotti S. (2020). Tryptophan in health and disease. Adv. Clin. Chem..

[60] Taleb S. (2019). Tryptophan Dietary Impacts Gut Barrier and Metabolic Diseases. Front Immunol..

[61] Lee S.M., Park H.Y., Suh Y.S., Yoon E.H., Kim J., Jang W.H., Lee W.S., Park S.G., Choi I.W., Choi I. (2017). Inhibition of acute lethal pulmonary inflammation by the IDO-AhR pathway. Proc. Natl. Acad. Sci. USA.

[62] Puccetti P., Grohmann U. (2007). IDO and regulatory T cells: A role for reverse signalling and non-canonical NF-kappaB activation. Nat. Rev. Immunol..

[63] Thomas T., Stefanoni D., Reisz J.A., Nemkov T., Bertolone L., Francis R.O., Hudson K.E., Zimring J.C., Hansen K.C., Hod E.A. (2020). COVID-19 infection alters kynurenine and fatty acid metabolism, correlating with IL-6 levels and renal status. JCI Insight.

[64] Rothhammer V., Quintana F.J. (2019). The aryl hydrocarbon receptor: An environmental sensor integrating immune responses in health and disease. Nat. Rev. Immunol..

[65] Stockinger B., di Meglio P., Gialitakis M., Duarte J.H. (2014). The aryl hydrocarbon receptor: Multitasking in the immune system. Annu. Rev. Immunol..

[66] Puccetti M., Paolicelli G., Oikonomou V., de Luca A., Renga G., Borghi M., Pariano M., Stincardini C., Scaringi L., Giovagnoli S. (2018). Towards Targeting the Aryl Hydrocarbon Receptor in Cystic Fibrosis. Mediators Inflamm..

[67] Moura-Alves P., Fae K., Houthuys E., Dorhoi A., Kreuchwig A., Furkert J., Barison N., Diehl A., Munder A., Constant P. (2014). AhR sensing of bacterial pigments regulates antibacterial defence. Nature.

[68] Grunewald M.E., Shaban M.G., Mackin S.R., Fehr A.R., Perlman S. (2020). Murine Coronavirus Infection Activates the Aryl Hydrocarbon Receptor in an Indoleamine 2,3-Dioxygenase-Independent Manner, Contributing to Cytokine Modulation and Proviral TCDD-Inducible-PARP Expression. J. Virol..

[69] Huai W., Zhao R., Song H., Zhao J., Zhang L., Zhang L., Gao C., Han L., Zhao W. (2014). Aryl hydrocarbon receptor negatively regulates NLRP3 inflammasome activity by inhibiting NLRP3 transcription. Nat. Commun..

[70] Agus A., Planchais J., Sokol H. (2018). Gut Microbiota Regulation of Tryptophan Metabolism in Health and Disease. Cell Host Microbe.

[71] Emami F., Vatanara A., Park E.J., Na D.H. (2018). Drying Technologies for the Stability and Bioavailability of Biopharmaceuticals. Pharmaceutics.

[72] Rosiere R., Berghmans T., de Vuyst P., Amighi K., Wauthoz N. (2019). The Position of Inhaled Chemotherapy in the Care of Patients with Lung Tumors: Clinical Feasibility and Indications According to Recent Pharmaceutical Progresses. Cancers.

[73] Fellner R.C., Terryah S.T., Tarran R. (2016). Inhaled protein/peptide-based therapies for respiratory disease. Mol. Cell Pediatr..

[74] Horava S.D., Moy K.J., Peppas N.A. (2016). Biodegradable hydrophilic carriers for the oral delivery of hematological factor IX for hemophilia B treatment. Int. J. Pharm..

[75] European Medicines Agency (2013). Assessment Report: Kineret.

[76] Kowalewski J., Ray A. (2020). Predicting novel drugs for SARS-CoV-2 using machine learning from a >10 million chemical space. Heliyon.

[77] Hathout R.M., Abdelhamid S.G., Metwally A.A. (2020). Chloroquine and hydroxychloroquine for combating COVID-19: Investigating efficacy and hypothesizing new formulations using Bio/chemoinformatics tools. Inform. Med. Unlocked.

[78] Puccetti M., Giovagnoli S., Zelante T., Romani L., Ricci M. (2018). Development of Novel Indole-3-Aldehyde-Loaded Gastro-Resistant Spray-Dried Microparticles for Postbiotic Small Intestine Local Delivery. J. Pharm. Sci..

[79] Tabas I., Glass C.K. (2013). Anti-inflammatory therapy in chronic disease: Challenges and opportunities. Science.

[80] Sahakijpijarn S., Moon C., Koleng J.J., Christensen D.J., Williams Iii R.O. (2020). Development of Remdesivir as a Dry Powder for Inhalation by Thin Film Freezing. Pharmaceutics.

